# Estimation of deracinated trees area in temperate forest with satellite images employing machine learning methods

**DOI:** 10.7717/peerj-cs.648

**Published:** 2021-08-19

**Authors:** Hideyuki Doi, Tomoki Hirai

**Affiliations:** 1 Graduate School of Information Science, University of Hyogo, Kobe, Japan; 2 Graduate School of Simulation Studies, University of Hyogo, Kobe, Japan

**Keywords:** Machine learning, CNN, SVM, Random forest, Disturbance, Forest, Landsat, Satellite image

## Abstract

Climate change can increase the number of uprooted trees. Although there have been an increasing number of machine learning applications for satellite image analysis, the estimation of deracinated tree area by satellite image is not well developed. Therefore, we estimated the deracinated tree area of forests *via* machine-learning classification using Landsat 8 satellite images. We employed support vector machines (SVMs), random forests (RF), and convolutional neural networks (CNNs) as potential machine learning methods, and tested their performance in estimating the deracinated tree area. We collected satellite images of upright trees, deracinated trees, soil, and others (*e.g*., waterbodies and cities), and trained them with the training data. We compared the accuracy represented by the correct classification rate of these methods, to determine the deracinated tree area. It was found that the SVM and RF performed better than the CNN for two-class classification (deracinated and upright trees), and the correct classification rates of all methods were up to 93%. We found that the CNN and RF performed significantly higher for the four- and two-class classification compared to the other methods, respectively. We conclude that the CNN is useful for estimating deracinated tree areas using Landsat 8 satellite images.

## Introduction

Trees in forests have various important roles in ecosystems (Bengtsson et al., 2000; Mori, Lertzman & Gustafsson, 2017), climate change (Newell & Stavins, 2000; Keenan et al., 2014), and human society, through their ecosystem services (Gamfeldt et al., 2013). Natural disturbances, such as wildfire, insect outbreaks, and windthrows have a serious impact on the functioning of forest ecosystems (Bengtsson et al., 2000; Mori, Lertzman & Gustafsson, 2017; Seidl et al., 2017). Trees are uprooted mostly due to windthrows (Ulanova, 2000). Additionally, the increased number of storms due to climate change has contributed to the deracination affecting the forest ecosystem (Seidl et al., 2017).

Earlier, it was necessary to conduct field surveys to estimate the deracinated trees area. However, currently, methods such as unmanned aerial vehicles (Getzin, Nuske & Wiegand, 2014) and light detection and ranging (LiDAR, Huang et al., 2013) are employed for the assessment. In addition, the satellite images, *e.g*., Landsat 8 and Sentinel-2 photographs, have been utilized in evaluating the forest disturbance, including the deracinated tree area (Lastovicka et al., 2020; Nguyen et al., 2020).

Using satellite images, an area with broader deracination compared to the aforementioned methods can be estimated. Furthermore, the use of global data from satellite images makes it possible to estimate the damage at any location and track the damage over time. However, even with satellite image analysis, the automatic estimation of the deracinated tree area is still limited. Recently, machine (deep) learning methods for image analysis have been developed for various purposes (Gulshan et al., 2016; Hird et al., 2017). We hypothesized that the machine learning method would be beneficial for the automatic estimation of the deracinated tree area.

In this study, we focus on the deracinated tree areas in temperate forests to estimate the forest disturbance. We aim to classify the deracinated trees using satellite images along with the mainstream machine-learning methods, such as support vector machine (SVM), random forest (RF) and convolutional neural network (CNN). These methods are the most widely used learning-based algorithms (Akar & Güngör, 2012). The SVM aims to find a linear discriminant function with maximum margin to separate each class. In SVM algorism, where the samples can be linearly separated, and then, the samples are classified in that space, and where they cannot be linearly separated, SVM transferred the data to a higher-dimensional space (Kaban & Diri, 2008). The RF is less sensitive to noise and subject to overfitting rather the other tree-based machine learning (Watts & Lawrence, 2008). The CNN is a more recent method and uses a multiple-level neural network, especially for image classification (*e.g*., Han, Liu & Fan, 2018). The objective of this study is to compare the performances of SVM, RF, and CNN methods to clearly distinguish the deracination area from the other areas.

## Materials & Methods

### Collection of image data

**Figure 1:**
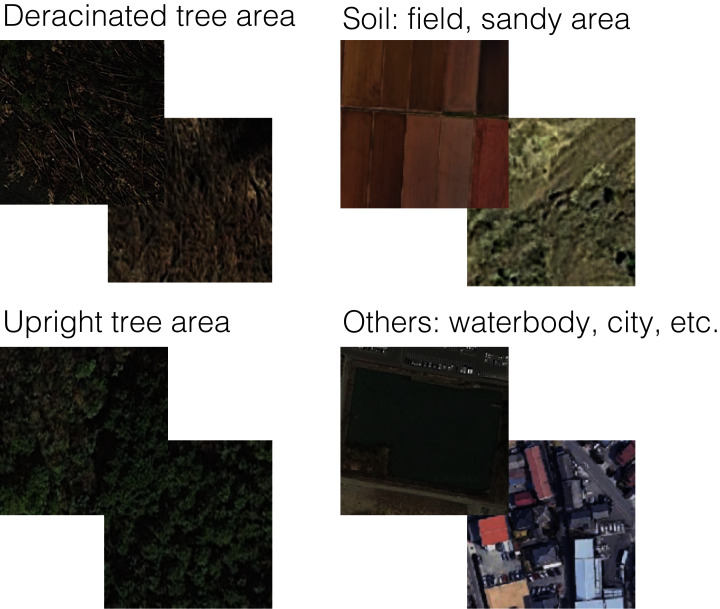
Example Landsat 8 satellite images for each test category in this study. All images from the Landsat 8 satellite were under CC-BY 4.0.

The training and test data for the learning algorithms were collected from satellite images using Landsat 8 images from the U.S. Geological Survey (USGS) Landsat Look Viewer (https://landsatlook.usgs.gov). Landsat 8 (formally, the Landsat Data Continuity Mission) was launched on February 11, 2013. The spatial resolution of Landsat 8 is 30 m per pixel. We set the following four training data categories: deracinated tree area, forest, soil (such as agricultural field), and others (*e.g*., waterbodies and cities). We physically searched the Landsat images for Japan during 2018, and collected 50 images (300 × 300 pixels) for each category (Fig. 1, all raw data in Supplemental Materials). We used k-fold cross validation method to train the machine learning with the training data and the remaining images as the test data, to evaluate the performance and rate of correct classification. We performed k-fold cross validation with “KFold” function in Python with (n_splits = 5, shuffle = True) options.

### Machine learning

In this study, we applied SVM, RF, CNN learning methods with ten replicated calculations with randomly selected training and test images. We performed analysis with either of the two sets: considering only deracinated or upright trees in the forests or considering four classes, *i.e*., deracinated tree area, upright trees, soil, and others (the raw data in Supplemental Materials). We performed the machine learning with adapting once for one batch (*i.e*., a set of the images).

### Support vector machine (SVM)

We performed principle component analysis (PCA) using Python ver. 3.8.0, and then, categorized the results using SVM. We calculated the ten PCs from training images. For SVM, we used the R package “kernlab”. The RBF kernel with “ksvm” function is used from the package and to set the parameters; σ = 0.01 and C = 1. Since SVM was originally used for two-class classification, we used the one-*vs*-rest and one-*vs*-one methods for the four-class validation. One-*vs*-rest is a heuristic method for using splitting the multi-class dataset into multiple binary classification algorithms for SVM multi-class classification. One-*vs*-one is the one-*vs*-one approach splits the dataset into one dataset for each class *versus* every other class for binary classification algorithms for SVM multi-class classification.

### Random forest (RF)

For the image analysis using RF algorithm, the R package “randomForest” was utilized. The number of trees was set to 500 as the default setting.

### Convolutional neural network (CNN)

We used a program from CNN programs available on GitHub, with train.py (GitHub: https://github.com/tensorflow/hub/blob/master/examples/image_retraining/retrain.py) for training, and label_image.py (GitHub: https://github.com/tensorflow/tensorflow/blob/master/tensorflow/examples/label_image/) for categorization. One technique in transfer learning is in which a model is trained in one domain, and then, used in another domain (Shin et al., 2016). This makes it possible to create training data with sufficient performance even when only a small amount of data is used for training. We used the Inception V3 model by Google Cloud (https://cloud.google.com/tpu/docs/inception-v3-advanced). This model is a CNN trained on a database of images called ImageNet and has an accuracy of over 78.1% on the ImageNet dataset (https://cloud.google.com/tpu/docs/inception-v3-advanced). Transfer learning was performed with Inception V3 using Tensorflow Hub (https://github.com/tensorflow/tensorflow), a library containing pretrained. We performed the different number of CNN pretraining permutations; 1, 10, 100, and 1,000, to check the number of permutations for the correct classification rate.

### Statistical analysis

We performed Welch’s paired t-test to assess the difference in correct classification rate s between the methods for two classes (upright and deracinated trees) and four classes (upright and deracinated trees, soil, and others). The significance level of α was set at 0.05. Since the multiple comparison is based on 3–4 methods, the *p*-value is correct for the Holm–Bonferroni method for multiple comparisons.

## Results

### Image analysis using SVM and RF

**Figure 2:**
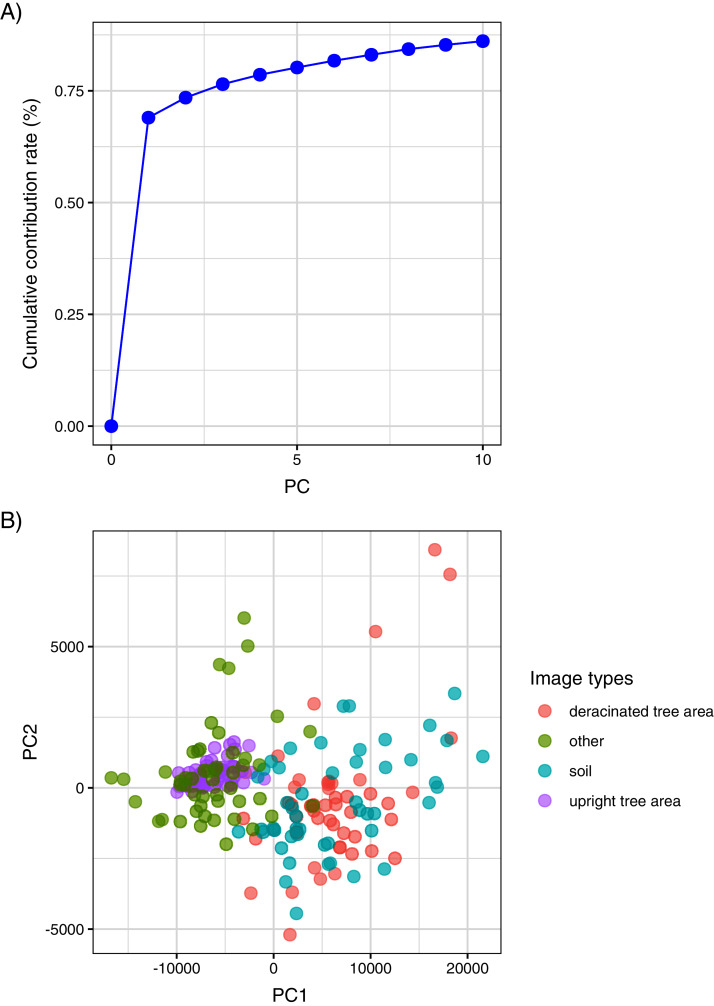
(A) Cumulative contribution rate of PCA with each PC value. (B) Ordination of PCA using PC1 and PC2 for deracinated (red) and upright (blue) tree area.

The contribution rate of each PC is shown as the cumulative contribution rate as presented in Fig. 2A, indicating that the cumulative contribution rate was over 70% at the time of the second PC. This implies that the image data were sufficient to perform SVM. From PCA, it was found that the boundary between the upright and deracinated trees can be drawn as illustrated in Fig. 2B; subsequently, SVM classifications can be performed. Therefore, we used the PCs extracted by PCA and classified them *via* SVM. We performed image analysis using an RF for number of RF trees from 100 to 1,000. However, no remarkable differences were noted. We used the default setting of 500 trees.

### Image analysis using CNN

**Figure 3:**
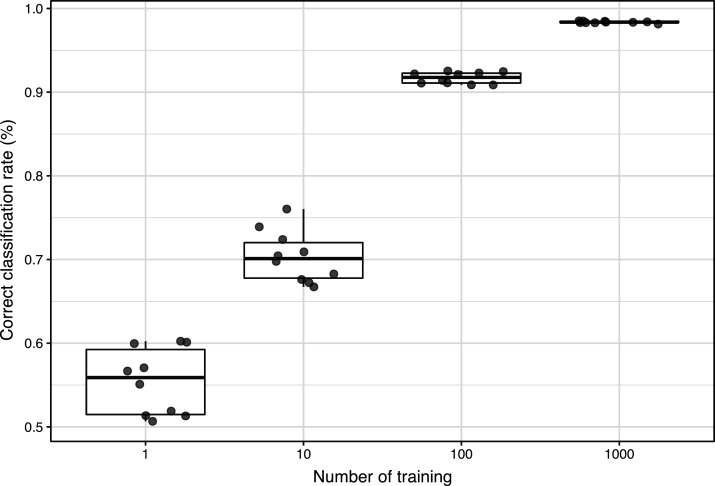
Correct classification rate of CNN with the number of learning times from 1 to 1,000. The boxes and bars in the box plots indicate median ± inter-quartiles and ±1.5 × inter-quartiles, respectively. The points represent individual data values. The characters indicate the significant differences (*p* < 0.05) resulted by Holm multiple comparison.

In machine learning methods using the images of deracinated trees and depending on the relationship between the number of times of learning and discrimination accuracy, the discrimination accuracy was approximately 50% when the number of learning times was 1. However, the correct classification rate increased as the number of learning times increased, and the rate was close to 100% when the number of learning times was 1,000 as indicated in Fig. 3. Multiple comparisons showed that the 1,000-times evaluation was significantly more accurate than the other permutations (Fig. 3, *p* < 0.0001, Holm multiple comparison).

### Correct classification rate of SVM, RF and CNN for deracinated or upright trees

**Figure 4:**
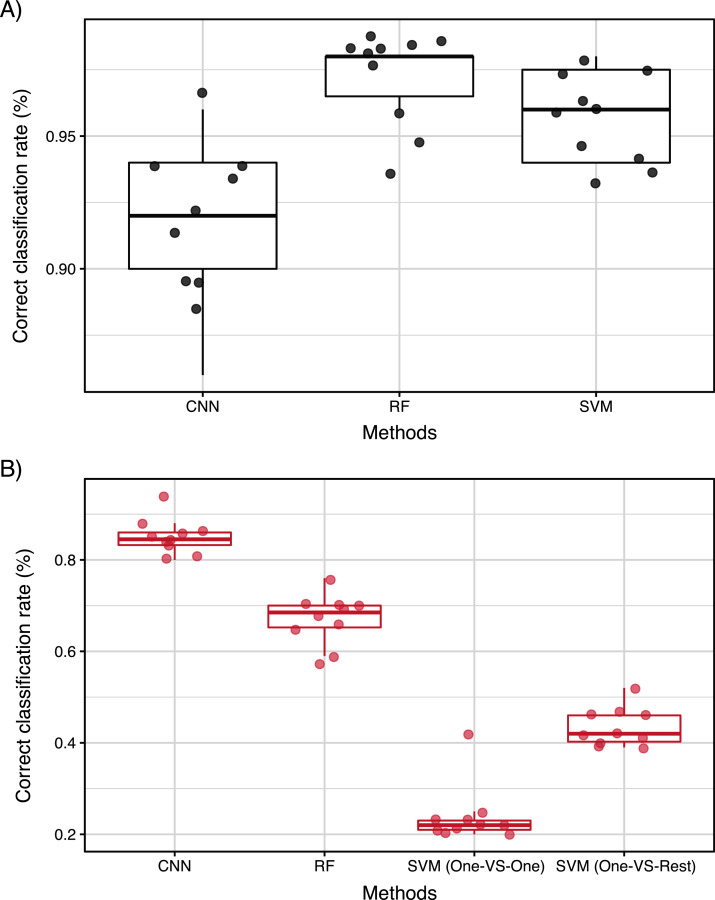
(A) Correct classification rate of SVM, RF, and CNN for two-class classification (B) correct classification rate of SVM, RF, and CNN for four-class classification. The boxes and bars in the box plots indicate median ± inter-quartiles and ±1.5 × inter-quartiles, respectively. The points represent individual data values. The characters indicate the significant differences (*p* < 0.05) resulted by Holm multiple comparison.

From the correct classification rate of SVM, RF, and CNN, it was found that all methods had a high correct classification rate above 85%. Multiple comparisons showed that the RF and SVM were significantly more accurate than CNN for the two-class classification as presented in Fig. 4A (*p* < 0.0001, Holm multiple comparison).

We compared the SVM (two methods: One-*vs*-Rest and One-*vs*-One), RF, and CNNs considering four-class classification as indicated in Fig. 4B. The comparison showed that the CNN was significantly more accurate in the four-class classification (Fig. 4B, *p* < 0.0001), unlike the two-class classification results. This result showed that CNN is more appropriate for four-class classification.

## Discussion

From the image analysis using SVM, RF and CNN, it was estimated that the accuracy of the CNN was significantly higher than that of RF and SVM in the four-class classification. Although in two-class classification, SVM and RF had better accuracy than CNN. The users can use these machine learning methods for various images with different geographies to extract the deracinated tree area. Previous machine-learning studies have shown that CNNs are more accurate than SVMs and RFs (*e.g*., Kussul et al., 2017), and the results of this study support the findings of previous studies on deracinated forest trees. Herein, we used transfer learning with a trained model for validation on CNN. However, its performance has been evaluated by other studies as well, and it was found to be suitable for discrimination in cases with little image data as in this study. Enhancing the learning by increasing the training data may result in higher discrimination by CNN. In this study, we used the limited image data (50 images for each category). Especially CNN needs to train with more image data, *i.e*., >1,000 images, while we found the higher performance of CNN than other methods with such limited data. We speculated that our CNN methods with Transfer Learning and k-fold cross validation would increase the performance. Further study needs to evaluate the performance of CNN with larger dataset.

We here tested the CNN for Landsat 8 images, but other satellite images such as Sentinel-2 or other Landsat (~7) images can be used for the analysis. Using the Landsat (approximately 7) images, we can evaluate the long-term forest dynamics, although we need to evaluate the spatial resolution of images between Landsat 7 and 8. The Landsat 8 images have some merits such as 30 m resolution permitting the estimation of the local scale of forest disturbance. In addition, Landsat 8 provides the opportunity for even more frequent observations of snow cover with a global median average revisit interval of 2.9 days. Therefore, we may be able to immediately estimate the forest disturbance caused by disasters, such as typhoons or hurricanes, in a broad area, for example, at the national level.

## Conclusions

In this study, we applied the three machine-learning methods, SVM, RF, and CNN to classify the deracinated tree area as an estimate of forest disturbance. We found CNN is the best machine-learning method for deracinated tree area evaluation by multiple-class classification. RFs are the best algorithm for two-class problem with limited data. Future work will investigate larger datasets and CNNs for limited data. Although this study only focused on deracinated trees, the results could also be applied to the disturbance estimation for other hazards using different types of training data, for example, by combining deracinated tree data and landslide data (*e.g*., Sameen, Pradhan & Lee, 2020). The results from this study could also be used for remote sensing for climate and land use (*e.g*., Ma et al., 2019), medicine (*e.g*., Işın, Direkoğlu & Şah, 2016), and health care (Khan & Yairi, 2018). Further improvement in discrimination accuracy would be desirable for estimating the forest damage against future disasters and climate change, as expected (Seidl et al., 2017). Especially, for post-disaster survey, we can obtain the images from Maxar dataset (https://www.maxar.com/open-data) to evaluate using machine learning. With testing the machine learning methods using diverse of forest types and tree species, the method can extend to evaluate global distribution of deracinated tree area.

## Supplemental Information

10.7717/peerj-cs.648/supp-1 Supplemental Information 1 Python codes for CNN and SVM performed in this study.

## Additional Information and Declarations

### Data Availability

The following information was supplied regarding data availability: All raw image data are available in Zenodo: Hideyuki Doi, & Tomoki Hirai. (2021). Estimation of deracinated trees area in temperate forest with satellite images employing machine learning methods [Data set]. Zenodo. DOI 10.5281/zenodo.4764161. The codes are available in the Supplemental File. We also used the codes available at GitHub: - https://github.com/tensorflow/hub/blob/master/examples/image_retraining/retrain.py. - https://github.com/tensorflow/tensorflow/blob/master/tensorflow/examples/label_image/.
